# A MR Fingerprinting Development Kit for Quantitative 3D Brain Imaging

**DOI:** 10.1002/jmri.70320

**Published:** 2026-03-28

**Authors:** Rasim Boyacioglu, Thomas Kluge, Guido Buonincontri, Wei-Ching Lo, Stephan Kannengiesser, Mathias Nittka, Arashdeep Kaur, Andrew Dupuis, Dan Ma, Chaitra Badve, Mark A. Griswold, Yong Chen

**Affiliations:** 1 Department of Biomedical Engineering, Case Western Reserve University, Cleveland, Ohio, USA; 2 Research & Clinical Translation, Magnetic Resonance, Siemens Healthineers AG, Erlangen, Germany; 3 Siemens Medical Solutions, Boston, Massachusetts, USA; 4 Department of Radiology, University Hospitals Cleveland Medical Center, Cleveland, Ohio, USA; 5 Department of Radiology, Case Western Reserve University, Cleveland, Ohio, USA; 6 Department of Neurosurgery, Duke University, Durham, North Carolina, USA; 7 Department of Biomedical Engineering, Duke University, Durham, North Carolina, USA

**Keywords:** accuracy, Bland–Altman, clinical translation, mapping, metastasis, MRF, quantitative MRI, relaxation, repeatability

## Abstract

**Background:**

Magnetic resonance fingerprinting (MRF) is an emerging quantitative imaging technique that enables multiparametric tissue characterization, but its adoption has been hindered by the complexity of data acquisition and post-processing. These technical and implementation challenges have limited its broader clinical deployment.

**Purpose:**

To develop a modular MRF Development Kit (MRFDK) that enables efficient sequence design, streamlined implementation, and real-time image reconstruction.

**Study Type:**

Prospective.

**Population:**

T_1_ and T_2_ relaxation phantom, nine volunteers (seven males and two females), five metastatic brain cancer patients.

**Field Strength/Sequence:**

3 T, MR Fingerprinting.

**Assessment:**

Accuracy of T_1_ and T_2_ quantification was estimated from phantom experiments. Manual ROIs were drawn on brain lesions and contralateral white matter for metastatic cancer patients.

**Statistical Tests:**

*t*-test, in vivo repeatability was calculated with Bland–Altman analysis on healthy volunteer scan-rescan data, significance level *p* < 0.01.

**Results:**

Phantom results showed high accuracy in T_1_ and T_2_ assessment, with absolute percentage differences of 3% for T_1_ and 5% for T_2_ compared to offline MATLAB reconstruction. In vivo scans of eight healthy subjects further demonstrated excellent repeatability (bias and agreement: 0.95% ± 1.85% for T_1_; 1.78% ± 5.08% for T_2_). In patients, metastatic lesions showed significantly higher T_1_ and T_2_ values (T_1_, 1474 ms; T_2_, 61 ms) compared to normal white matter (T_1_, 913 ms; T_2_, 38 ms). With integrated B_1_ correction, all T_1_ and T_2_ maps were available for visualization within 1 min post-MRF scan, enabling immediate image assessment.

**Data Conclusion:**

A modular MRF development package enabling efficient 3D acquisition and rapid inline reconstruction was developed and evaluated in this study.

**Level of Evidence:**

1.

**Technical Efficacy:**

Stage 2.

## Plain Language Summary

Magnetic resonance fingerprinting (MRF) is an MRI technique that can measure multiple tissue properties simultaneously. Although many technical advances have been made, its use in the clinic has been limited due to the lack of a unified, fully integrated system. In this study, we developed a modular framework called MRFDK to simplify MRF sequence design and implementation in clinical settings. MRFDK combines all necessary steps for data acquisition and post-processing, enabling robust performance and easy deployment across scanners and sites. Tests in phantoms and human subjects further demonstrated the high accuracy, repeatability, and clinical utility of MRFDK.

## 1 ∣ Introduction

MRI is inherently limited by its inability to measure tissue properties quantitatively, which is important for objective and scanner-independent diagnosis and treatment monitoring [1-3]. Magnetic Resonance Fingerprinting (MRF) is an emerging quantitative imaging framework that enables simultaneous measurement of multiple tissue parameters in a single MRI scan [4]. Earlier studies have demonstrated the potential of MRF in improving clinical diagnosis across various pathophysiologies [5-11].

Compared to conventional quantitative MRI methods, MRF introduces unique features in both data acquisition (e.g., pseudorandomized acquisition, non-Cartesian spiral readout) and post-processing (dictionary-based pattern matching), largely enhancing its performance in tissue assessment. However, these innovations also pose substantial challenges in development, prototyping, and scanner deployment of the technique. As a result, current MRF implementations are limited with fixed field-of-views (FOVs) and spatial resolutions, reducing the flexibility in prescribing an optimal imaging setting for each subject. MRF post-processing is time-consuming and mostly conducted offline, hindering prompt visualization of acquired results and integration with standard clinical workflows.

In this study, we aimed to develop and validate a modular MRF Development Kit (MRFDK) for rapid and efficient MRF prototyping to achieve (1) flexible and efficient 3D whole-brain MRF acquisitions with adjustable FOVs and resolutions, (2) embedded MRF dictionary computation, and (3) rapid, inline MRF post-processing. The developed MRFDK package has been officially released for quantitative 3D brain imaging and provides a practical platform for large-scale clinical validation and translation of MRF. Beyond brain applications, this versatile platform also has the potential to advance quantitative MRI research across more organs and pathological conditions.

## 2 ∣ Methods

The study was approved by the local institutional IRB and was HIPAA compliant. For all the healthy volunteers, informed consent was obtained prior to the MRI exams. The developed method was also evaluated by imaging five patients with a variety of metastatic brain tumors. For these patients, informed consent was waived with the approval of a specialized IRB protocol, and the MRF scan was added to the patients’ routine clinical MRI exam before contrast administration.

The workflow contains four major steps, including sequence definition, dictionary generation, data sampling, and post-processing. Among the workflow steps, MRF sequence and dictionary are generated and fixed offline prior to the MRF exam, whereas MRF reconstruction occurs online in sync with the flexible acquisitions at the scanner. The technical improvements brought by MRFDK are: (1) simplified MRF sequence generation, (2) MRF container structure, (3) acquisition and sampling flexibility, and (4) efficient online reconstruction. Each improvement is detailed in the corresponding subsections below.

### 2.1 ∣ Preparation of MRF Sequence

**FIGURE 1 ∣:**
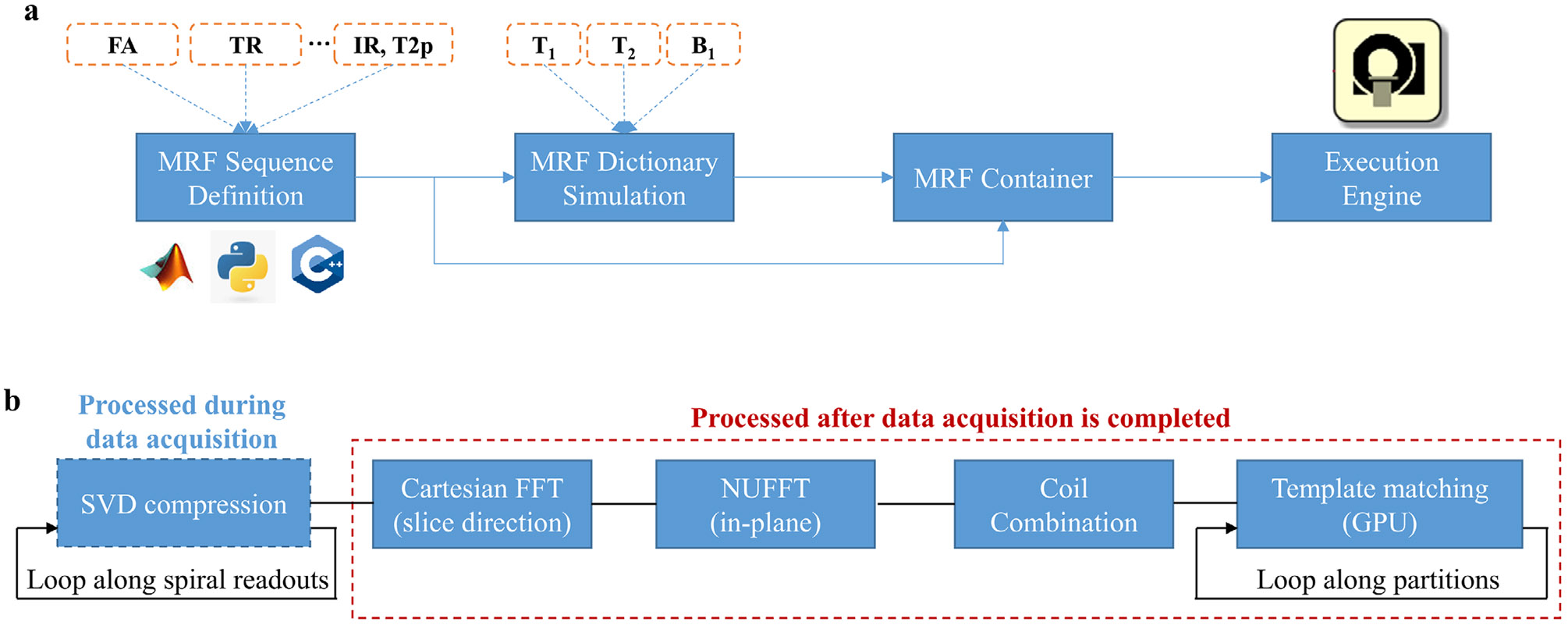
(a) MRFDK pipeline to generate MRF sequence definition, MRF dictionary, and finally a MRF container file to be copied and used on an execution engine on the MR scanner for the data acquisition. A variety of acquisition parameters (flip angles (FA), repetition times (TR), etc.) and preparation modules (inversion recovery (IR), T2-preparation (T2p), etc.) can be defined in the framework. (b) Online MRF post-processing pipeline. Singular value decomposition (SVD) was first performed to accelerate data processing. For 3D MRF with the stack-of-spirals trajectory, the SVD compression was performed immediately after MRF data for each spiral readout was acquired. GPU-enabled template matching was further implemented to accelerate the tissue mapping step.

To implement an MRF experiment, a pulse sequence definition must first be designed based on the target tissue properties. In this study, we focused on demonstrating the framework for quantitative T_1_ and T_2_ mapping, which is the most common application of the MRF framework [12]. Beyond T_1_ and T_2_ mapping, the MRFDK package also supports the quantification of other types of tissue properties, such as T_2_* [13] and water diffusion [14, 15]. The MRF sequence definition file is a high-level and simple template to prescribe only the core elements of an MRF sequence. The sequence definition can be generated using various development environments, including C++, MATLAB, python (Figure 1a), and two examples are provided in Figures S2 and S3. The user, based on their programming environment choice, has the flexibility to define MRF acquisition patterns (flip angles (FA), repetition time (TR), etc.) as well as several other key components, including the RF pulses, echo position of each readout, spoiling moments, and a feasible echo time (TE) and baseline TR value. The essential sequence objects (RF pulses, gradients), timing and order of events, and necessary checks are prepared by the framework based on the prescription in the definition file at runtime at the scanner. Based on the MRF sequence definition, an MRF dictionary is generated. A single file called MRF container is created for each application with the sequence definition and dictionary and transferred to the MRI scanner for direct MRF measurement and reconstruction. Users cannot alter the content of MRF containers at the scanner but can vary certain decoupled sequence parameters that essentially are realizable with the core MRF parameters (TR, TE) and still comply with the MRF dictionary in the MRF container.

### 2.2 ∣ MRF Dictionary Generation

Based on the sequence definition and target tissue properties, Bloch equation simulations are performed to generate the corresponding MRF dictionary. The computation is implemented in C++ and optimized with parallel processing across multiple CPU cores to enhance efficiency. The ranges and step sizes for each tissue property of interest are imported using user-specified text files. Signal simulation can also incorporate preparation modules such as inversion recovery and T_2_-preparation modules, along with an additional dimension of the B1 field.

### 2.3 ∣ Data Acquisition

An MRF execution engine, that is, interpreter sequence, was pre-installed on MR scanners to run MRF sequences and utilize the dictionaries within the MRF containers (each corresponding to a different acquisition sequence). This engine allows selection from a list of MRF containers, enabling seamless deployment of various MRF acquisition protocols. The interpreter sequence allows tuning of sequence parameters which are not bound by the fixed parameters in the sequence definition and dictionary. The customizable sequence parameters are FOV, resolution, slice thickness, gradient specifications (for peripheral nerve stimulation purposes), and sampling, acceleration, and interpolation in 2D and 3D. Unlike prior MRF implementations with fixed FOVs and resolutions, the MRFDK framework also integrates an online spiral design framework, allowing customization of FOV and matrix size for each MRF scan. When the user changes FOV or image matrix, the spiral is designed at the scanner from scratch rather than using a precomputed look-up table. The designer module within the framework first evaluates the target spatial coverage and sampling demand in k-space, then builds a physically feasible spiral under the scanner’s gradient amplitude and slew-rate limits. Additionally, the inner and outer k-space regions can be assigned different sampling densities, and these densities are directly configured by the user in the UI. After this re-design, a new set of gradient samples and interleaf rotation angles is generated, and those samples define the updated k-space path used by reconstruction. Gradient delay correction was applied online for each spiral trajectory during image reconstruction. The pipeline carries a Tan–Meyer style correction model [16] alongside the trajectory during acquisition-reconstruction handshake. The model captures axis-specific timing offsets and eddy-current compensation terms, so reconstruction can correct the effective k-space location from where the nominal trajectory is prescribed to where it was actually traversed. The correction is orientation-aware (patient/slice geometry is included), so the delay/eddy current model is applied consistently in physical gradient axes and then mapped to the acquisition frame. This largely improves the flexibility in prescribing MRF scans, matching conventional Cartesian-based, T_1_- or T_2_-weighted MR exams. For 3D MRF acquisitions, a stack-of-spirals trajectory was used for volumetric encoding. As in previous studies [17, 18], all MRF time points were acquired consecutively for each slice phase encoding stack, that is, partition, and the same acquisition pattern was then repeated sequentially across all the partitions. To accelerate 3D scans, an interleaved undersampling strategy along the slice-encoding direction was implemented [17] and an undersampling factor up to four can be selected from the sequence card. Based on the selected undersampling factor, multiple partitions are acquired in an interleaved manner in one repetition of the time frames. In addition, partial Fourier and interpolation in the slice-encoding direction are enabled.

### 2.4 ∣ Image Reconstruction and Tissue Mapping

Another key feature of the MRFDK framework is an efficient inline reconstruction pipeline implemented using the local image reconstruction unit on the scanner (Figure 1b). For each MRF spiral readout, partial compression along the temporal dimension based on singular value decomposition (SVD) [19] was performed while the next spiral arm was acquired. SVD compression matrix is pre-calculated during MRF dictionary generation and is also stored within the MRF container structure. This approach not only reduces the number of images to be reconstructed, but also effectively utilizes data sampling time to improve post-processing efficiency. This process was repeated until the MRF data for all the partitions were acquired. A Cartesian FFT was then performed along the partition direction, followed by 2D NUFFT for each slice and coil combination. Finally, GPU-accelerated template matching was implemented to accelerate the tissue-mapping step. B1 correction was automatically performed if B1 maps were acquired prior to the MRF scan [20] by matching each voxel with the portion of the dictionary corresponding to its B1 value. A single preceding B1 map is utilized by all the following MRF scans in a session.

### 2.5 ∣ Phantom Validation

All MR scans were performed on a 3 T scanner (MAGNETOM Vida, Siemens Healthineers, Forchheim, Germany). We first validated the accuracy of the proposed method in vitro using the standardized MRI relaxation time phantom developed by the National Institute of Standards and Technology (NIST) [21].

MRF sequence definition file was created in MATLAB. The imaging parameters included: FOV, 25 × 25 cm^2^; matrix, 256 × 256; slice thickness, 2 mm; 72 slices; 920 TRs/time frames; FA, 1°–60° (adopted from a previous study [18]); TR, 8.7 ms; waiting time between partitions, 2 s; acceleration along the slice-encoding direction, 2; unbalanced SSFP readout with 2π dephasing; total scan time, ~6.5 min. Only one inversion recovery module with an inversion time of 21 ms was applied at the beginning of data acquisition of each partition, and no T_2_-preparation module was included. Leveraging the online spiral design method, a total of 40 spiral arms were generated to meet the Nyquist criterion based on the specified FOV and resolution. As in prior MRF studies [17, 18], only one spiral arm was acquired per plane, and the spiral arms were increasingly rotating by 9° across time frames. A vendor product B1 mapping acquisition of ~20 s was also applied before the MRF acquisition to enable online B1 correction. The quantitative T_1_ and T_2_ relaxation time maps were obtained during online reconstruction. The map quality was compared to the maps obtained offline using MATLAB reconstruction based on the same raw data file. Region-of-interest (ROI) analysis was performed to extract T_1_ and T_2_ values for all the vials in the T_1_ and T_2_ layers of the NIST phantom, respectively. The extracted values were further compared to the reference values provided by NIST.

### 2.6 ∣ In Vivo Brain Imaging

We applied the MRFDK framework using the same container from phantom validation for in vivo 3D neuroimaging with healthy volunteers and patients. Both B1 and MRF scans with the same settings as in the phantom experiment were acquired for each subject using a 20-channel head coil and all the MRF maps were reconstructed online. For one healthy subject (male, 48 years old), the same acquisition was repeated with a 32-channel head coil. In addition, the same MRF container was applied for one healthy subject (male, 22 years old) to acquire 3D brain T_1_ and T_2_ maps with a different FOV of 30 × 30 cm^2^ and matrix size of 256 × 256 (in-plane resolution, 1.2 mm).

### 2.7 ∣ Evaluation of Repeatability for Brain T_1_ and T_2_ Quantification

Repeatability of MRFDK was tested using a scan/rescan protocol and automated regional Bland–Altman analysis [18]. Nine healthy volunteers (2 females, age: 42.5 ± 14.6 years, one discarded due to motion) were scanned twice with the 20-channel head coil and the 3D MRFDK protocol used for the phantom scans. Synthetic magnetization-prepared rapid gradient echo (MPRAGE) contrast weighted images were generated from T1 maps to obtain nonlinear transformations to the Montreal Neurological Institute (MNI) space using FMRIB (Functional Magnetic Resonance Imaging of the Brain) Software Library (FSL) [18, 22]. Anatomical regions from the Harvard-Oxford cortical and subcortical structural atlases were projected to individual subject spaces based on the transformations and eroded to avoid partial volume effects. Bland–Altman plots for multiple regions were created to show bias and agreement between scan and rescan MRFDK across the whole brain.

### 2.8 ∣ Characterization of Brain Metastases

The clinical utility of 3D MRFDK protocol was explored in a pilot, retrospective study of metastatic brain tumors. A total of five patients with new and non-treated tumors (4 males, age: 65 ± 12 years) were scanned with MRFDK and circular ROIs were drawn on a total of 10 lesions to extract T_1_ and T_2_ values to characterize brain metastatic lesions. The ROI was drawn by a clinical radiology fellow (A.K.) with two years of experience in neuroradiology.

### 2.9 ∣ Statistical Analysis

All the data acquisition and analysis were performed independently at our institution, and the vendor had no control of the acquired data, analysis, or results. Mean and standard deviations were calculated based on the ROI analysis for both phantom and in vivo experiments. To evaluate the accuracy of the quantitative T_1_ and T_2_ measurements obtained with MRFDK, the mean absolute difference was calculated for the water phantom data processed using online MRFDK reconstruction and offline MATLAB reconstruction. Students *t*-test was performed to compare T_1_ and T_2_ values obtained from normal white matter versus brain metastatic lesions in the pilot clinical study. *p* value less than 0.01 was considered statistically significant.

## 3. ∣ Results

### 3.1 ∣ Phantom Validation

**FIGURE 2 ∣:**
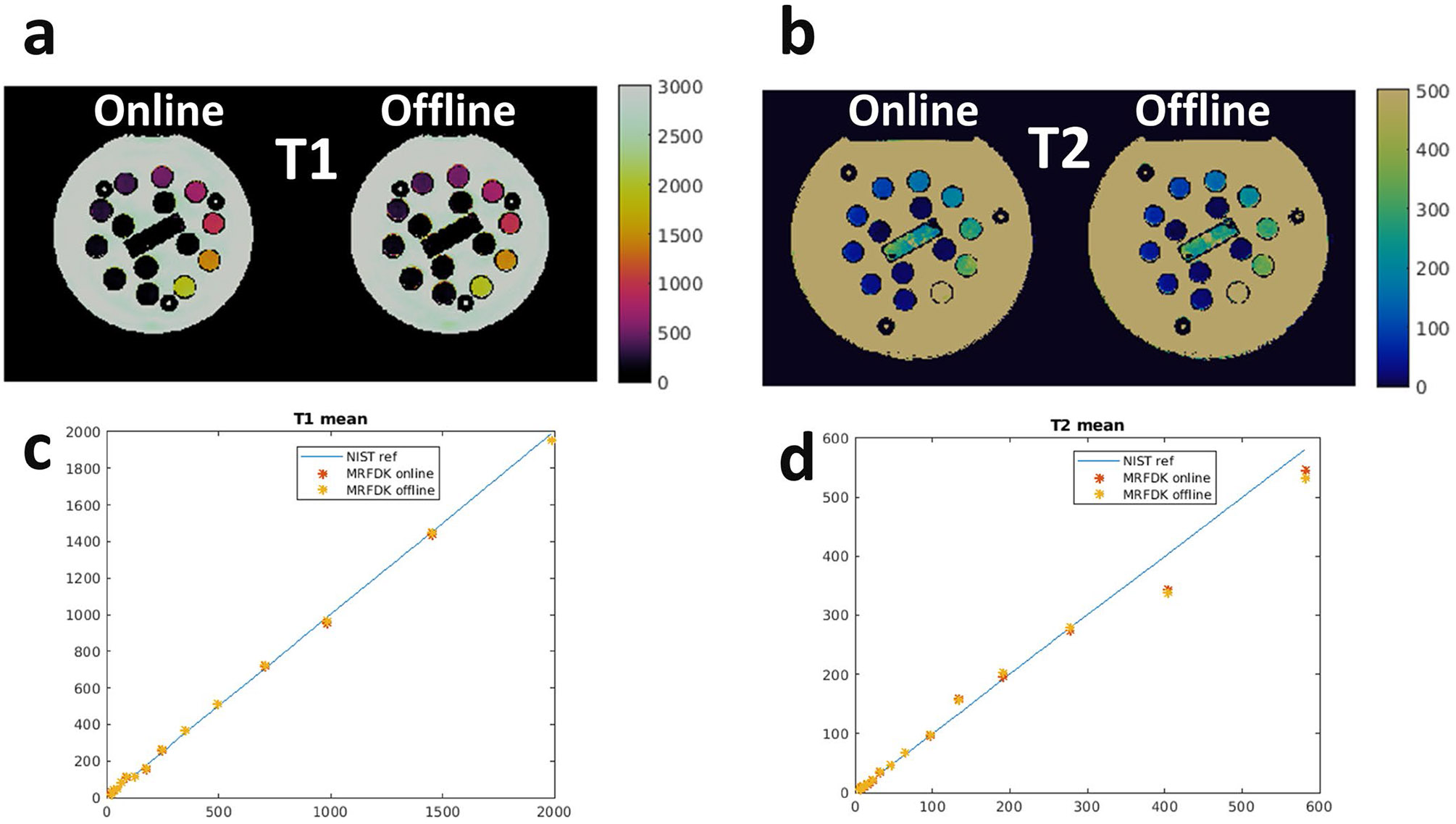
Phantom validation. Representative T_1_ (a) and T_2_ (b) maps for the NIST phantom with online and offline reconstruction. Correlation of the MRF-derived T_1_ (c) and T_2_ (d) values with the reference values provided by NIST.

Using the NIST phantom, a close agreement in quantitative T_1_ and T_2_ assessment was observed between the online MRFDK reconstruction and offline MATLAB reconstruction from the same raw dataset (Figure 2). The mean absolute difference between the MRFDK and MATLAB results was 5 ± 5 ms (3%) for T_1_ and 3 ± 3 ms (5%) for T_2_. Additionally, the MRFDK results showed good agreement with the reference T_1_ and T_2_ values provided by NIST (Figure 2).

### 3.2 ∣ Flexible In Vivo MRF Measurement With B1 Correction

**FIGURE 3 ∣:**
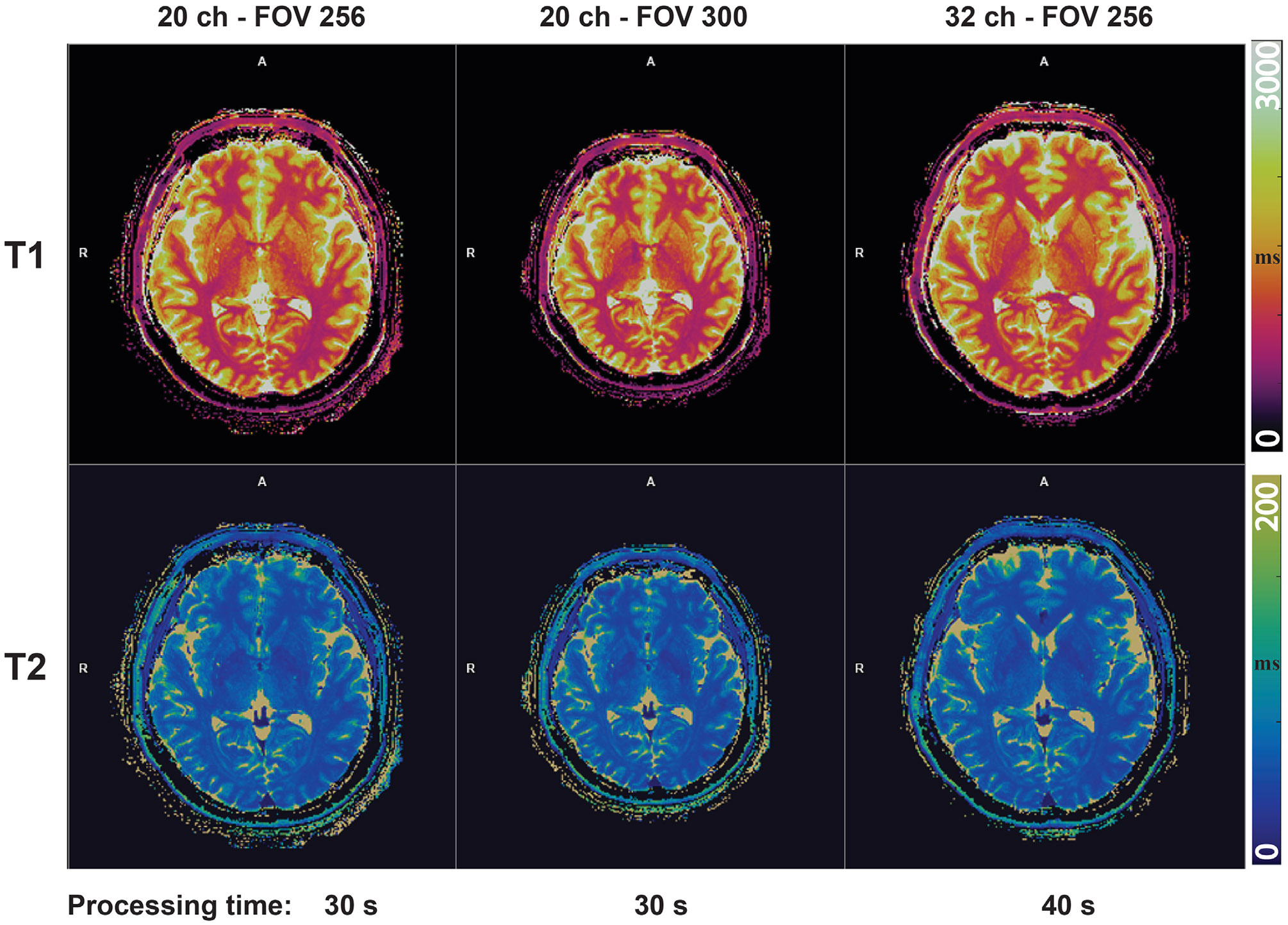
Representative MRF maps obtained with a healthy volunteer with different FOVs (25 and 30 cm) and head coils (20 channel and 32 channel head coils). Direct screen capture from the console is presented to illustrate the map quality achieved through online reconstruction. All the MRF maps were available for visualization on the host computer within 40 s after the scan was completed.

**FIGURE 4 ∣:**
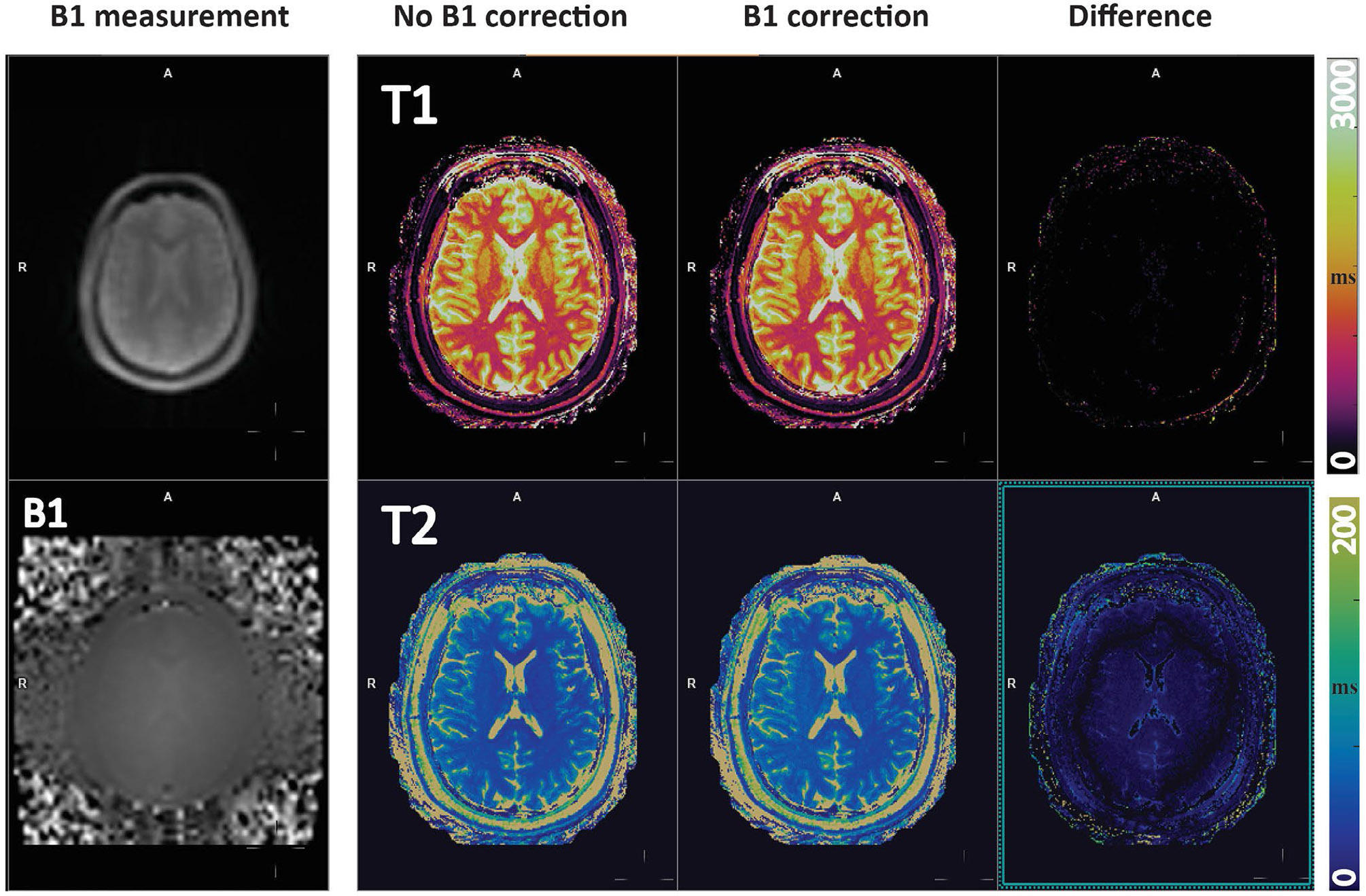
3D brain MRFDK with B1 correction. Volumetric B1 mapping was performed before the 3D MRF acquisition. MRF reconstruction with and without B1 map is shown in the middle two columns. The corresponding difference maps are shown in the right column. More uniform T_2_ maps were observed after the B1 correction, matching the measured B1 map shown in the first column.

Figure 3 presents a direct screen capture of the MRF T_1_ and T_2_ maps from the scanner’s host computer, obtained from the same subject with two different FOVs (25 and 30 cm). For both settings, the tissue property maps obtained with MRFDK using a 20-channel head coil were available for visualization on the host computer 30 s after the scan was completed. The processing time increased by 10 s when switching to a 32-channel head coil (Figure 3). Figure 4 shows a screen capture of an example obtained with and without B1 correction, along with the T_2_ difference map, which shows good agreement with the acquired B1 map.

### 3.3 ∣ Repeatability of T_1_ and T_2_ Assessment With MRFDK

**FIGURE 5 ∣:**
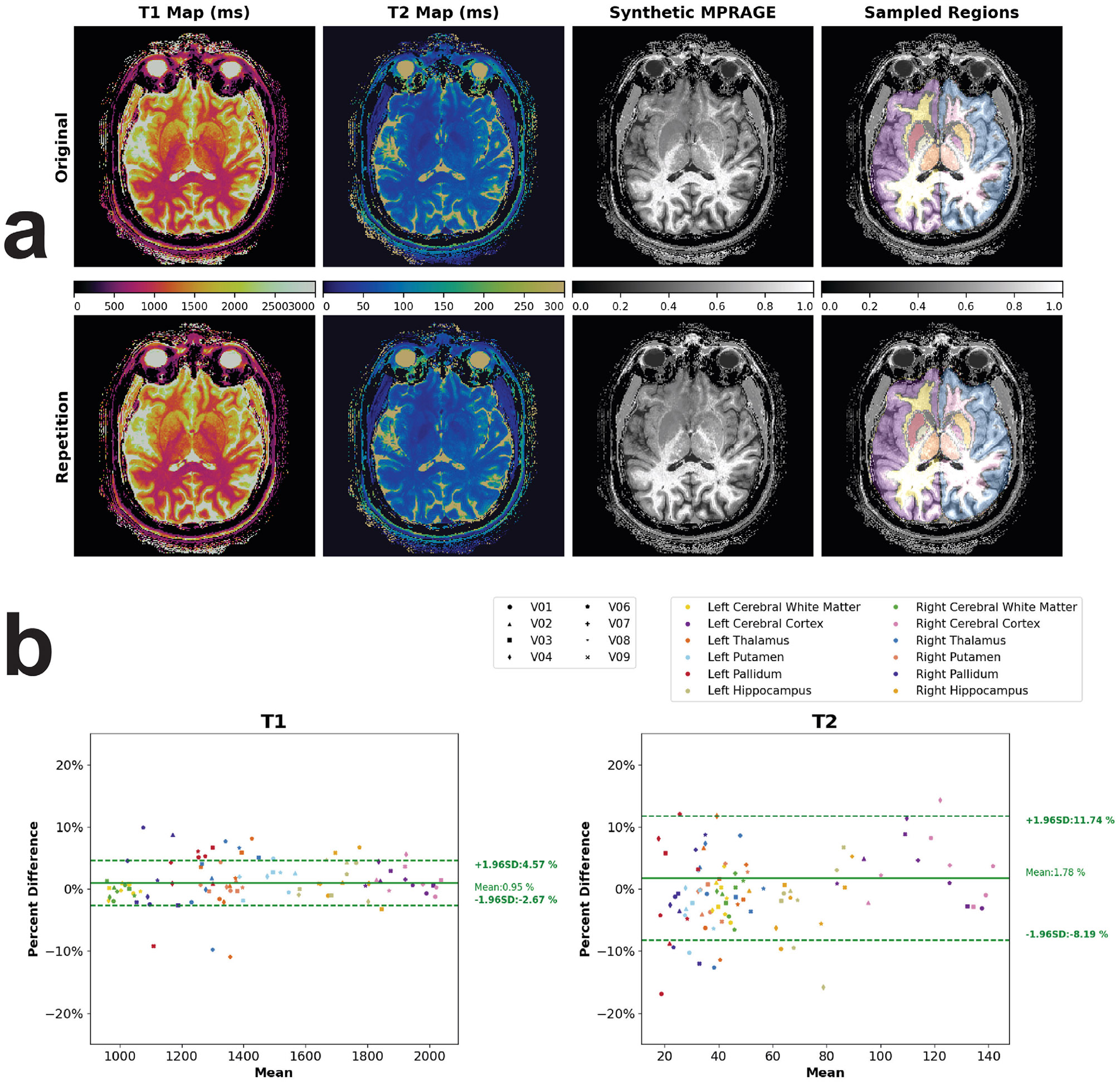
(a) Scan (top) and rescan (bottom) repeatability study quantitative maps, synthetic MPRAGE images (only used for registration) and anatomical regions from a representative subject. (b) Bland–Altman plots illustrate the repeatability of 3D MRFDK protocol. Different subjects and regions were plotted with different shapes and colors, respectively. Aggregate bias ± agreement values weighted by region volume are shown on the top half of the plots. Both T_1_ and T_2_ have little to no bias (0.95% and 1.78%) and good agreement between scan and rescan (1.85% and 5.08%).

Figure 5a depicts the MRFDK maps, the synthetic MPRAGE images for registration and the anatomical regions that feed into the Bland–Altman analysis for the repeatability study. 3D MRFDK demonstrated excellent repeatability with the corresponding bias and agreement: 0.95% ± 1.85% for T_1_ and 1.78% ± 5.08% for T_2_. The Bland–Altman plots in Figure 5b also show a uniform distribution of repeatability across multiple brain regions and subjects.

### 3.4 ∣ Pilot Clinical Evaluation of MRFDK

**FIGURE 6 ∣:**
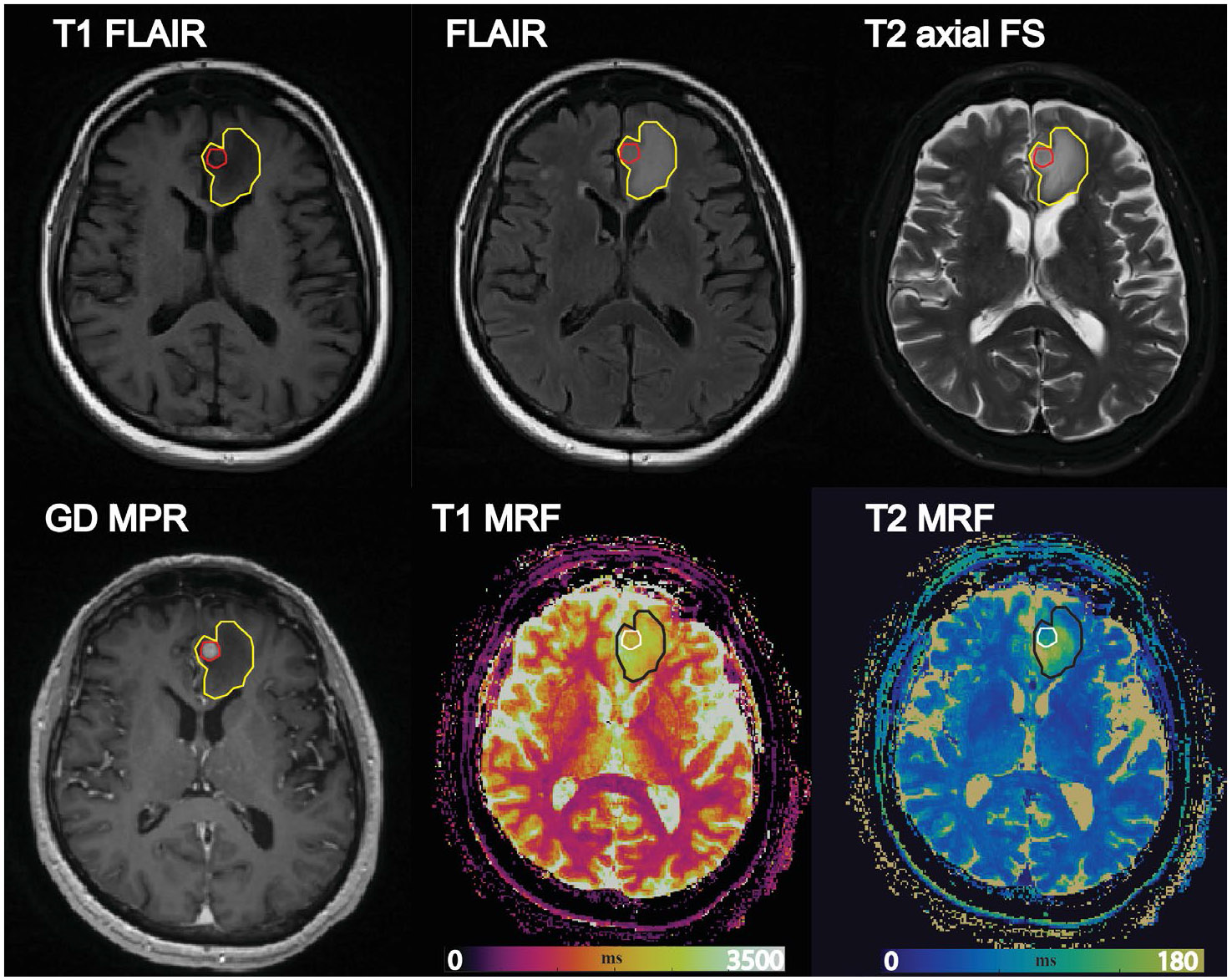
Representative results obtained from a 73-year-old male patient with a metastatic gastric tumor in the frontal cortex. MRFDK T_1_ (bottom middle) and T_2_ (bottom right) maps are shown together with pre-contrast T1-weighted, T2-weighted, and FLAIR, and post-contrast T1-weighted images. The tumor and surrounding edema are contoured for MRFDK maps and weighted images.

Representative T_1_ and T_2_ maps obtained from a patient (male, 73 years old) with metastatic gastric lesions are presented in Figure 6. The mean T_1_ and T_2_ values of all 10 lesions were 1474 and 61 ms, respectively, both higher compared to the values obtained from normal appearing white matter regions (T_1_, 913 ms; T_2_, 38 ms; *p* < 0.01; Table 1).

## 4 ∣ Discussion

In this study, we introduced the MRFDK framework for efficient MRF design, acquisition, and online processing. High-quality brain tissue maps are displayed on the console in less than 1 min after scan completion. The phantom and in vivo results demonstrated the quantitative measurements with MRFDK are within adequate accuracy and repeatability.

Through academic and vendor collaboration, the developed MRFDK modularized multiple components of the MRF framework, including sequence definition, dictionary generation, and data acquisition and processing. The in vivo repeatability of quantitative brain imaging achieved by MRFDK is smaller or on the order of a single dictionary step (4% step size) and comparable to another 3D MRF reproducibility study focused on quantitative brain imaging [18]. This high precision of MRFDK establishes clear boundaries of random variation and provides thresholds for detecting T_1_ and T_2_ changes across various disease conditions. Our pilot clinical study, which focused on brain metastatic lesions, also demonstrates results consistent with the literature findings where higher T_1_ and T_2_ values were observed in metastatic lesions compared with normal appearing white matter [23], highlighting the potential of MRFDK in clinical applications. Reproducible and quantitative MRF maps provide an opportunity for radiologists to define new biomarkers, particularly for cancer diagnosis, when combined with existing traditional MRI features.

Unlike traditional MRF implementations with rigid protocols and offline processing, MRFDK offers a highly flexible framework that enhances adaptability, accuracy, and workflow integration. Due to ease of use, similar features in UI, and immediate DICOM availability, patients and radiographers observe typical scan conditions and protocol functionality. Through the MRFDK execution engine, users can select a container from a list of MRFDK protocols designed to target various applications or body parts, for example, an MRF sequence [24] with low FAs and T_2_-preparation pulses to minimize sensitivity to B1 inhomogeneities (Supporting Information) versus the MRF sequence demonstrated in this study with relatively higher FAs for enhanced SNR. In addition, the container structure ensures the appropriate dictionary is linked during dictionary matching, preventing mismatches that could potentially occur during offline MRF processing. Easier and faster dissemination of MRF sequences is also possible in a traceable manner as the MRF container structure ensures the same MRF sequence, SVD compression, and dictionary are deployed when distributed to other scanners and sites. On the acquisition side, flexibility from the spiral design framework coupled with various acceleration options allows for inline testing of the sampling limits for a given MRF sequence. In terms of efficiency, compared to offline reconstructions taking up to 30 min depending on the number of slices and coils, MRFDK returns quantitative map DICOMs to the scanner console in 30–40 s by carrying out certain reconstruction steps during image acquisition.

## 5 ∣ Limitations

First, since the baseline TR is specified in the sequence definition and used for dictionary generation, it is fixed in a container and can partially limit the FOV and matrix size. An alternative solution would be to prepare a few containers with different TRs and choose the most efficient one based on acquisition details. Second, motion detection and correction are not included in this version of MRFDK, which is important in imaging challenging populations such as pediatric subjects. In future implementations, motion detection and correction, inline B0 and B1 correction, more advanced capabilities for mapping additional (to T1 and T2) contrasts and more advanced tissue analysis such as partial volume analysis [25], could be integrated to the NRFDK framework. In addition, we aim to validate the reproducibility and stability over different sites with a larger clinical study.

## 6 ∣ Conclusion

We introduced and evaluated the MRFDK framework for efficient 3D brain MRF acquisition and rapid online reconstruction. Using phantom, volunteer, and patient studies, MRFDK demonstrated accurate T_1_ and T_2_ quantification with high in vivo repeatability. The integration of online reconstruction and B1 correction enables quantitative maps to be available within 1 min following the MRF acquisition. Together, the developed package streamlines the MRF development process and has great potential to facilitate broader clinical evaluation and adoption of this advanced quantitative MR imaging technique.

## Supplementary Material

Supplementary

Additional supporting information can be found online in the Supporting Information section. **Figure S1:** To demonstrate the versatility of MRFDK, another 3D MRF protocol using IR/T_2_-preparation modules and golden-angle spiral encoding was also implemented and evaluated on one healthy subject for quantitative brain imaging. **Figure S2:** Code snapshot for the generation of sequence definition file for 3D MRF with a single inversion pulse. This sequence was used for the phantom validation and in vivo experiments in the main manuscript. **Figure S3:** Code snapshot for the generation of sequence definition file for 3D MRF with multiple inversion and T2-prep pulses. This sequence was used for the example demonstrated on Figure S1.

## Figures and Tables

**TABLE 1 ∣:** Aggregate T_1_ and T_2_ values (means ± standard deviations) extracted from five patients with a total of 10 newly diagnosed metastatic lesions.

	Lesions	White matter	Gray matter	CSF
T_1_ (ms)	1474 ± 243	913 ± 89	1398 ± 209	3841 ± 59
T_2_ (ms)	61 ± 7	38 ± 3	65 ± 13	590 ± 13

*Note:* The values for both lesions and normal appearing brain regions (white matter, gray matter, and cerebrospinal fluid (CSF)) are included. Both T_1_ and T_2_ in the metastatic lesions are higher than the values obtained in normal appearing white matter (*p* < 0.01).

Abbreviation: CSF, cerebrospinal fluid.

## Data Availability

The MRF interpreter sequence has been provided as a research software package by Siemens Healthineers. The MRF development kit is part of the Siemens software development tools and is available via collaboration contracts with Siemens Healthineers.
