# Supplementary material for: A MR Fingerprinting Development Kit for Quantitative 3D Brain Imaging
Source: J Magn Reson Imaging. Author manuscript; Available in PMC 2026 Apr 29. (PMC13122508; doi:10.1002/jmri.70320)
Supplement: Supplementary [file NIHMS2165368-supplement-Supplementary.docx]

**Supplemental Materials**

**
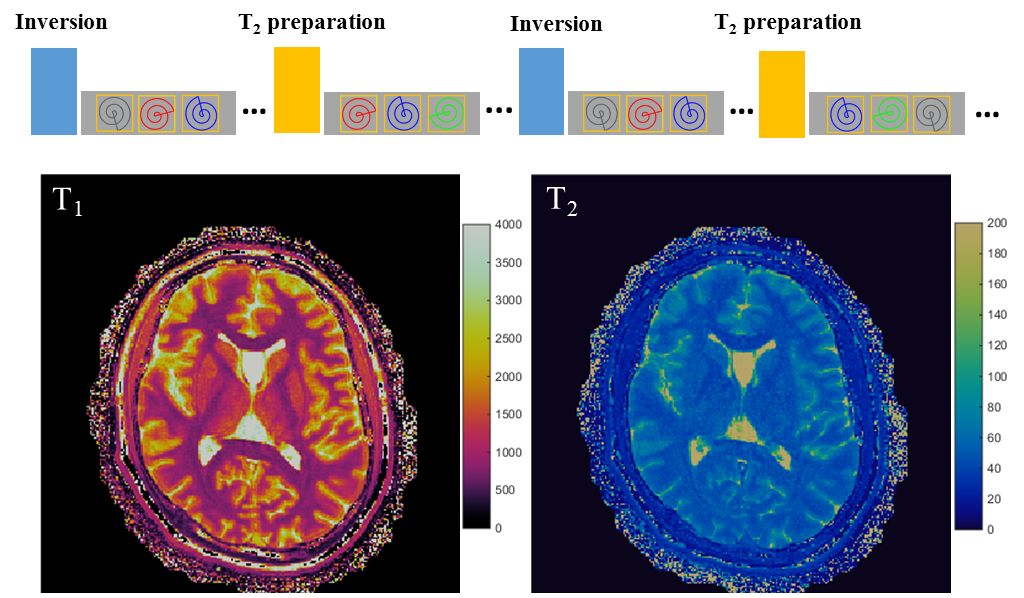
**

**Figure S1:** To demonstrate the versatility of MRFDK, another 3D MRF protocol using IR/T_2_-preparation modules and golden-angle spiral encoding was also implemented and evaluated on one healthy subject for quantitative brain imaging.

**
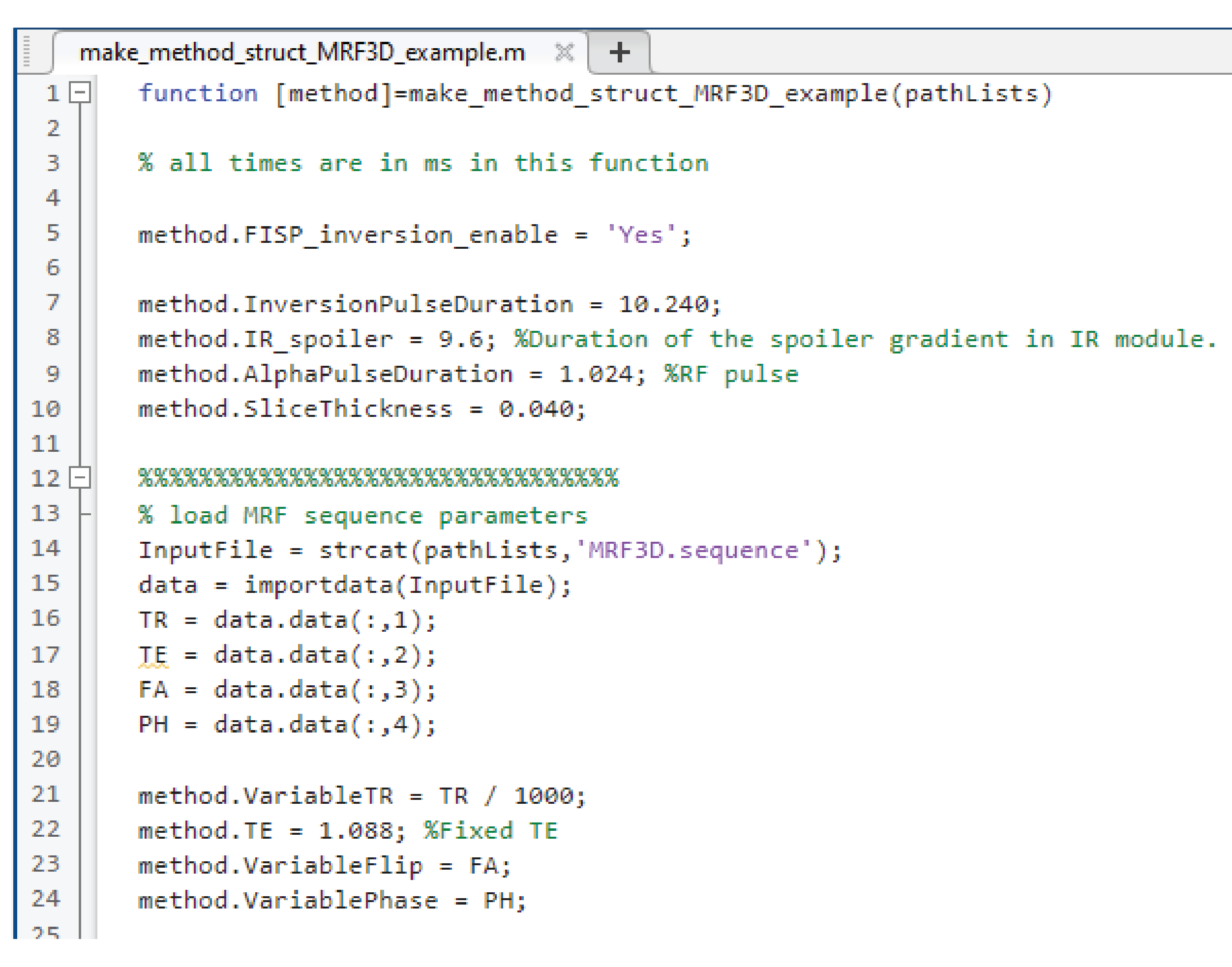
**

**Figure S2:** Code snapshot for the generation of sequence definition file for 3D MRF with a single inversion pulse. This sequence was used for the phantom validation and in vivo experiments in the main manuscript.


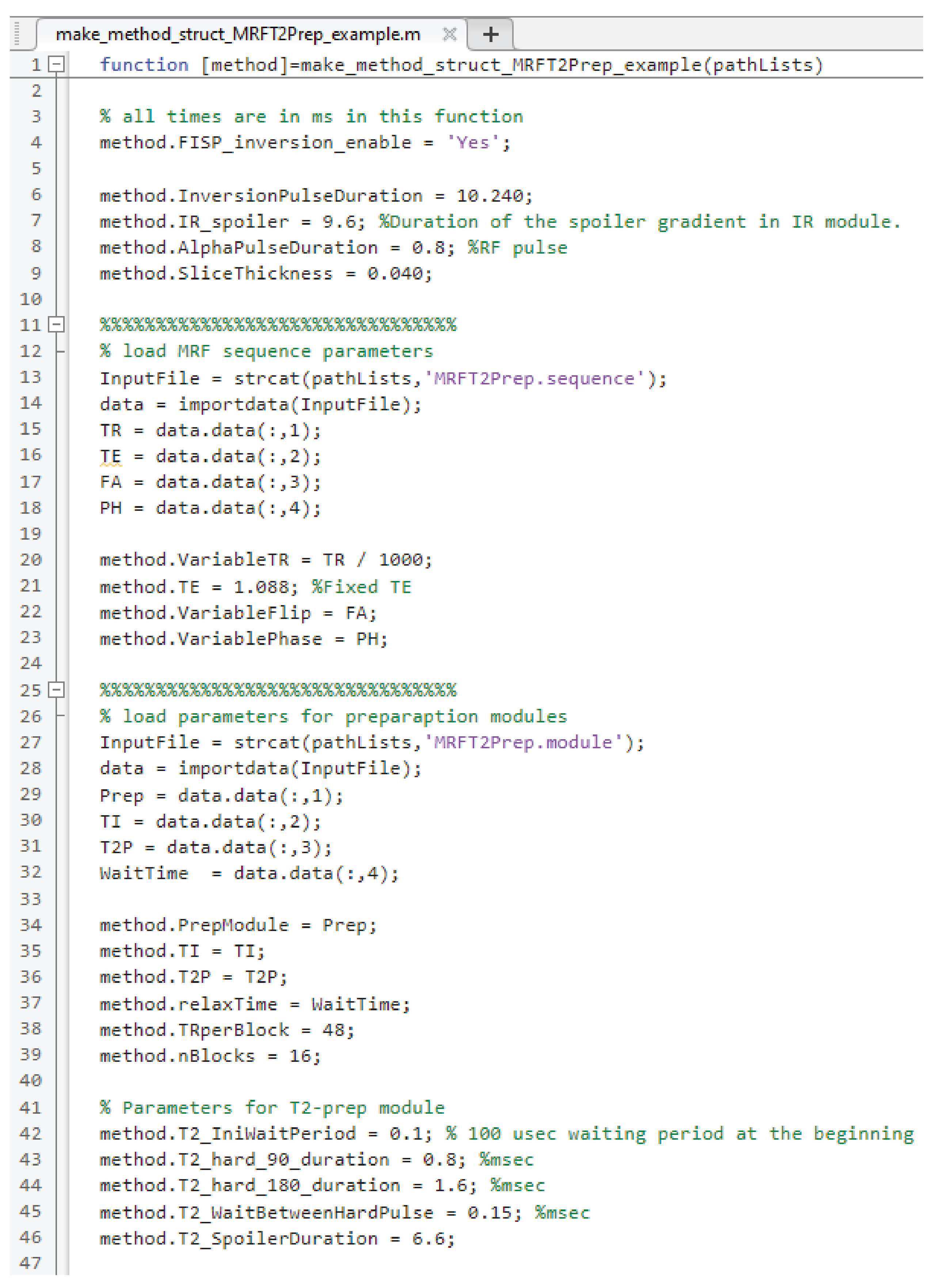


**Figure S3:** Code snapshot for the generation of sequence definition file for 3D MRF with multiple inversion and T2-prep pulses. This sequence was used for the example demonstrated on Figure S1.
